# Integrated mental health screening for obstetric fistula patients in Mali: From evidence to policy

**DOI:** 10.1371/journal.pone.0238777

**Published:** 2020-09-04

**Authors:** Melissa H. Watt, Thuy-vi Nguyen, Cheick Touré, Demba Traoré, Jennifer Wesson, Joy Noel Baumgartner

**Affiliations:** 1 Duke University, Durham, NC, United States of America; 2 University of Utah, Salt Lake City, UT, United States of America; 3 IntraHealth International, Inc., Bamako, Mali; 4 IntraHealth International, Inc., Chapel Hill, NC, United States of America; University of California San Francisco, UNITED STATES

## Abstract

**Background:**

Obstetric fistula significantly impacts women’s mental health and well-being. Routine screening for mental health in fistula repair programs can be a gateway to link patients to services, and can produce routine data to inform programmatic investments. This study observed the integration of a mental health screening program into an obstetric fistula repair program in Mali, with two specific objectives: 1) to describe the social and mental health well-being of women presenting with obstetric fistulas in Mali, and 2) to document the impact of the mental health screening pilot on policy change in Mali.

**Methods:**

Seven fistula repair campaigns were conducted between June 2016 and May 2017. All individuals presenting for fistula repair completed a mental health assessment at intake, including a depression screener (PHQ-9) and an assessment of psycho-social impacts of fistula. The depression screener was repeated three months following inpatient discharge. Findings were shared with stakeholders in Mali and impacts on policy were documented.

**Results:**

Of 207 women who presented for fistula repair, 167 patients completed the mental health assessment at surgical intake, and 130 patients repeated the screener at 3-month follow-up. At intake, 36.5% of women had moderate or severe depression, decreasing to 16.9% at follow-up. The mean depression score differed significantly by timepoint (9.14 vs. 6.72, p <0.001). Results were shared in a report with stakeholders, and consultations with the Mali Ministry of Health. As a result of advocacy, mental health was a key component of Mali’s National Fistula Prevention and Treatment Strategy (2018–2022).

**Conclusion:**

The high prevalence of depression in Malian fistula patients underscores a need for more robust mental health support for patients after surgery. Data on mental health from routine screening informs community reintegration strategies for individual patients, elevates the overall quality of care of fistula repair programs by addressing patients’ holistic health needs, and contributes to evidence-informed decision-making and data-driven policy change within the larger health system.

## Introduction

Obstetric fistula is a debilitating complication of childbirth that is typically the result of prolonged and unrelieved obstructed labor [1]. An estimated 2–3 million women worldwide have an obstetric fistula, though this figure is likely an underestimate due to the low visibility and awareness associated with the condition [2]. Obstetric fistula primarily affects rural women in low-income countries who have limited access to emergency obstetric services. Women who suffer from an obstetric fistula have urinary and/or fecal incontinence, which has severe impacts on both their physical and mental health [3]. The mental health burden of fistula patients has been documented in sub-Saharan Africa and South Asia. Prevalence of depression has varied widely across studies, ranging from 23% to 93% [4–8], and other psychological symptoms, including post-traumatic stress disorder, somatic complaints, and maladaptive coping, have been documented [7]. In addition, the stigma associated with a fistula can impact women’s ability to participate fully in social and productive activities. Enacted stigma leads to abandonment and dissolution of personal relationships, and anticipated stigma can lead women to avoid public spaces and social gatherings [9–11]. Isolation and ostracization, combined with internalized stigma, can lead to feelings of shame, anxiety and depression [12].

Obstetric fistula is surgically treatable for most women, but many barriers exist to prevent the timely repair of a fistula [13]. In addition to shortages of trained providers and facilities, social, cultural and psychological barriers combine to delay women’s presentation at care [13]. Although successful repair of a fistula can have a significant impact on mental health symptoms, distress can continue even after fistula repair surgery, especially when there is residual incontinence [14–17]. While fistula surgical repair programs vary in their components, there is little published data on the types of mental health care available to this patient population. Fistula patients are often not screened for mental health disorders and the few studies that have assessed mental health conditions in fistula patients report that conditions often persist after surgery, yet are seldom addressed [6, 18].

Surgical repair for an obstetric fistula, which requires an extended residential stay at a health facility, offers a window of opportunity to identify accumulated distress from a fistula, and to provide targeted psychosocial support and treatment. Timely mental health screening for mental health disorders and psychosocial stressors as part of patient intake enables health care providers to make linkages to treatment, is associated with better treatment outcomes [19], and provides an opportunity for monitoring mental health status and psychosocial well-being post-repair.

Increasingly, there have been global calls to address maternal mental health and to consider opportunities for integrating mental health care into maternal and child health programs [20, 21]. The goal of the present study was to observe the integration of a mental health screening program into an obstetric fistula repair program in Mali via a collaboration between a non-governmental organization (NGO) and a research institution, with two specific objectives: 1) to describe the social and mental health well-being of women presenting with obstetric fistulas in Mali, both at time of presentation to care and three months post-repair, and 2) to describe the process by which the mental health screening pilot program resulted in a policy change in Mali. The second objective is unique in that it responds to calls for more mutual learning and reflective practice between NGOs and researchers, and calls for better illuminating the processes of knowledge translation and pathways to policy impact [22–24]. The lessons learned in implementing a mental health screening pilot in a fistula repair program in Mali might be used to improve the policy and practice of fistula care more broadly.

## Materials and methods

### Overview

This study was an observational study of a fistula repair program in Mali. Between June 2016 and May 2017, mental health screening was integrated into seven fistula repair campaigns throughout the country. Data on clients’ self-reported mental health symptoms at clinical intake and 3 months post-discharge were extracted from individual medical records. This study and its program related outcomes are closely aligned with WHO’s Special Initiative for Mental Health (2019) which calls for strategic actions to encourage more local mental health policies that promote the scaling of integrated, quality mental health care for vulnerable groups within community and general health settings [25]. Ethical approval for the study was received by the Duke University Institutional Review Board.

### Setting

In Mali, the USAID estimated that of the 4 million women of reproductive age, 1,000 women develop an obstetric fistula each year [26]. The burden of fistula in Mali is likely underestimated, especially in rural populations, and there is a shortage of health care workers who are qualified to provide fistula care [27].

This study was conducted during the 2014–2018 USAID Mali-funded Capacity Building for Fistula Treatment and Prevention Project (“Fistula Mali Project”), led by IntraHealth in collaboration with the Mali Ministry of Health and Public Hygiene and national non-governmental organizations (NGOs) [28]. A strategic aspect of the project is making surgical repair of fistula accessible across the country, and in particular in the remote, rural areas where fistula is most prevalent. In the context of limited surgical repair resources, IntraHealth trained surgical teams in the priority geographic zones and organized campaigns to identify women with fistula and provide surgical treatment. Between June 2014 and November 2018, the program trained 25 fistula surgeons, conducted 36 fistula repair campaigns throughout the country, and completed 1,214 surgeries.

### Procedures

Seven fistula repair campaigns were conducted in four locations in Mali (Kayes, Koulikoro, Sikasso, and Gao) between June 2016 and May 2017. Female patients were identified by local NGOs who had established presence in the surrounding communities. The NGOs facilitated the transportation of the women to the surgical repair location. All female patients who presented for fistula repair were assessed using a clinical intake form and a brief screening form to assess for mental health distress. The mental health assessment (PHQ-9, see Measures) was completed by peer educators (PEs) from local NGO partners who had been trained to administer the tool and provide follow up. In the event that a patient indicated moderate or severe mental health distress, the PE provided psychosocial support, monitored the patient’s well-being during the surgical in-patient period, and made referrals within the hospital, if appropriate.

Approximately three months after discharge, patients were revisited, either in their communities or at a local hospital, and the clinical assessment and mental health screening were repeated. All participants who met the criteria for moderate or severe depression received additional counseling or psychosocial support from the PEs or referral to the local NGO or clinic. In the case of severe depression or suicidality, the client was referred to the previously-identified point of contact for fistula at each site for further support.

### Measures

The clinical intake form included demographics (e.g., age, education, marital status, number of children) and clinical indicators (e.g., type of fistula, number of past fistula surgeries). Depression was assessed using the PHQ-9, a 9-item measure that assesses the presence and severity of depressive symptoms in the past two weeks [29]. The PHQ-9 measure was translated from French [30] into the relevant national languages (Bambana, Peulh, Soninké, Sonrhai, Malinké). Reliability of the scale in the sample was strong (α = 0.79). Item scores were summed to produce a total depression score ranging from 0 to 27, and levels of depression were categorized using standard cut-offs: mild (score 5–9); moderate (10–14); moderately severe (15–19); and severe (≥ 20) (α = 0.79). Patients were also asked 14 questions about social impacts of living with a fistula, with each question having a yes/no response. Questions included whether a woman was isolated from her family or community, whether she felt that she brought shame to her family, and whether she felt that she didn’t deserve to live. The 14 impacts were chosen based on common psychosocial impacts women may have experienced as a result of living with a fistula. These items were identified based on input from local collaborators and a literature review [7, 9, 16, 17].

### Analysis

Programmatic data from the paper-based clinical intake forms and mental health assessments were entered in a spreadsheet in Mali, and de-identified data were shared with the research team for analysis. Data were exported to SPSS (ver. 24) and cleaned and scored prior to analysis. Demographic and clinical data about the patient population were described. Baseline and 3-month follow-up PHQ-9 scores, as well as social impacts and PHQ-9 scores, were compared using two-tailed t- tests.

### Dissemination of findings and documentation of practice and policy impacts

The screening results were assembled into a report and disseminated to key stakeholders who served on regional- and national-level steering committees for fistula (e.g., Ministry of Health and Public Hygiene [MoH], other partners supporting the fistula program, and Malian NGOs). IntraHealth met with the MoH to use the findings to advocate for the deployment of qualified human resources in psycho-social care (psychologists, psychiatrists and lay health workers) at decentralized facilities in the country. Given the role that IntraHealth plays in health system strengthening in Mali, we were able to observe and document the impact that the screening data had on a national policy document and subsequent programmatic approaches for fistula programs in Mali [31]. Meeting notes from the relevant steering committees, recollections from our Mali-based IntraHealth colleagues, and programmatic monitoring activities served as the data sources for describing these policy and programmatic impacts [32].

## Results

### Description of the population

Table 1 describes the patient population. Many women were presenting for repeat surgeries of an unrepaired fistula.

**Table 1:** Description of the population (n = 207).

Age	34.4 (SD: 14.1)
No formal education	80%
Married	72%
Age at first marriage	15.9* (SD: 18.7)
Age at first pregnancy	17.2 * (SD: 8.8)
Any living children	59%
Repeated surgery	71%

* = reported as means

### Depressive symptoms

Of the 207 women who presented for care, 167 patients (81%) responded to the PHQ-9 at surgical intake, and 127 patients (76%) responded to the PHQ-9 at a 3-month follow-up. Women who returned to follow up were older than those who did not return for follow up (p < .05), but there was no statistically significant difference between the two groups in baseline depression scores. At baseline, 36.5% met criteria for moderate or severe levels of depression (Table 2), and the patient population had a mean PHQ-9 score of 9.14 (SD 4.82, Range 1–22). Three months later, 16.9% of individuals who completed the follow-up assessment met criteria for moderate or severe depression (Table 2), and this population had a mean score of 6.72 (SD 4.25, Range 0–23). Table 3 compares the change in PHQ-9 scores among the 127 patients who had both baseline and follow-up assessments. On average, PHQ-9 scores decreased by 2.20 points (SD 5.18) at the 3-month follow-up (p <0.001). Despite this considerable decrease in depression symptomatology, it is notable that 40.0% of women at follow-up continued to endorse some thoughts of suicidal ideation in the past two weeks (item 9 of the PHQ-9).

**Table 2:** Depression severity of participants at baseline and 3 months.

Depression Severity (PHQ-9 score)	Baseline (n = 167)	Follow-up (n = 127)
No Depression (0–4)	15.6%	30.8%
Mild Depression (5–9)	47.9%	52.3%
Moderate Depression (10–14)	19.1%	12.3%
Severe Depression (15–27)	17.4%	4.6%

**Table 3:** Comparison of PHQ9 scores among patients with a baseline and follow-up assessment (n = 127).

	Mean (SD)	t	p-value
Baseline	8.79 (4.36)	4.78	< .001
Follow-up	6.59 (4.22)		

### Social impacts of fistula

Table 4 presents the social impacts that were reported by women, sorted by decreasing frequency. On average, women reported a mean of 7.5 impacts (SD 0.20, Range 2–14). The total number of impacts endorsed was not significantly correlated with depression score (r = 0.070, p = 0.370). One impact, however, “feeling punished by God”, was significantly associated with depression score at baseline (8.74 vs. 10.75, *t =* -2.18, p = 0.031). No impact was significantly associated with a change in depression score between baseline and 3 months.

**Table 4:** Endorsement of fistula impacts (n = 167).

Impact	% Reported
Disturbing fistula memories	92%
Stillborn child	85%
Feeling punished by God	80%
Feeling hopeless about the future	78%
Financial dependence	69%
Feeling like they brought trouble to their family	68%
Feeling like they didn’t deserve to live	64%
Avoided leaving the house	47%
Gossiped about	43%
Divorce/abandonment	25%
People stopped visiting	16%
Made to sleep/eat separately	15%
Hidden from the community by their family	12%
Stopped attending community events	10%

### Impact of findings on policy

The Mali MoH received the results of the mental health screening data in early 2018. The Mali country director of IntraHealth (CT) met with MoH partners (e.g., technical fistula partners, representatives of civil society, and MoH program managers who participated in the regional and national steering committees) to discuss the mental health findings and related policy implications. The Mali IntraHealth team indicated that steering committee members valued having Mali-specific mental health data (versus sharing mental health data from other sub-Saharan African countries) as this local data was particularly important for engaging stakeholders and making the case that there was a mental health treatment gap that needed to be addressed. These discussions facilitated the introduction of what the stakeholders called the *Depressive Assessment Approach for Fistula Victims* in the new National Fistula Standards in Mali, which called for universal mental health screening for fistula patients. This approach was then integrated into Mali’s National Fistula Prevention and Treatment Strategy (2018–2022), a national policy document developed with multi-stakeholder input [31].

## Discussion

At presentation to fistula care, over one-third of women (36.5%) presented with moderate or severe levels of depression. These levels are higher than populations living with other chronic and stigmatized health conditions, such as HIV, but lower than other fistula populations, where the prevalence of depression ranges from 47–74% [4, 6, 8, 33, 34]. Although the severity of depression had decreased significantly by the 3-month follow-up, moderate or severe depressive symptoms persisted for many patients (16.9%), and a large proportion (40.0%) continued to endorse some thoughts of suicidal ideation in the past two weeks. This data underscores the need for mental health support both prior to and following fistula repair surgery, especially since residual incontinence following surgery is associated with persistent mental health distress [14, 35]. Continued poor mental health could hamper community reintegration efforts that are typically part of fistula care and treatment programs [36–38].

Though the number of psychosocial impacts endorsed was not significantly associated with depression, it is notable that an overwhelming majority of women reported having disturbing memories of the fistulas (92%), feeling punished by God (82%), and feeling hopeless about the future (79%). The endorsement of these social impacts parallels other studies which have documented the wide-ranging psychosocial impacts of living with a fistula [9, 36, 39]. Stigma associated with obstetric fistulas might play a role in exacerbating these social impacts, as enacted and anticipated stigma can prompt women to avoid going into public spaces and attending social gatherings, or cause strained social relationships with spouses, family, and friends [9–11]. The only social impact significantly associated with depression is “Feeling punished by God,” which may reflect negative religious coping as an expression of depressive symptoms [40]. The stigma also permeates into religious spheres, as women with fistulas are often isolated from their places of worship or prohibited from practicing their faith [17, 41].

This study demonstrates the feasibility of integrating mental health screening into fistula care, as 81% of women presenting for fistula repair surgery received baseline depression screening, and of these women, 76% were reached for follow-up depression screening. Timely screening of mental health conditions is associated with better treatment outcomes, and screening can identify gaps in care delivery to fistula patients [42–44]. In addition, data collected via screening can have significant impacts on policy and practice when used as local evidence to engage key stakeholders, as it can demonstrate a need for services such as counseling. Further, as the fistula repair pathway is a rare point of contact between women in low- and middle-income countries and health systems, the pathway presents a rare window of opportunity for service provision, especially when women are recovering from their surgery [45]. Thus, it is recommended that all women presenting for fistula repair surgery are screened for depression, and that counseling or treatment be made available for women who screen positively. Given the limited availability of trained mental health providers in LMICs, task shifting interventions to nursing staff or community health workers is warranted [14, 46]. In this case study, screening data helped to convince the Malian government of both the need and opportunity to address mental health during fistula surgical care campaigns. The result was the integration of mental health into Mali’s national strategic plan for obstetric fistula and, hopefully, higher quality care for patients in the future. We note that this national policy level impact most likely came to fruition not only because of the data itself but because IntraHealth played the role of technical expert, implementer and *knowledge broker* for communicating these data-based results—a role that has been documented as critical in many situations for translating evidence into policy [47]. The university partners on this project were not involved in the policy advocacy beyond summarizing the study results in a brief report accessible to non-researchers, but the IntraHealth partners were able to present the data in clinical context and further engage in discussions for how a revised policy could be put into practice given their deep professional experience implementing the fistula repair campaigns.

This study used programmatic data from fistula repair campaigns; as implementation research, the study is limited due to restrictions in design and data available. First, our measure for depression (PHQ-9) was not validated for local languages in Mali, and there is a possibility that it may not adequately capture the construct of depression in this population. However, our confidence in the measure is increased by the strong reliability coefficient (α = 0.79), and the fact that the PHQ-9 is one of the most widely used depression screeners across Africa and has been validated across a range of African languages [48–50]. Second, our study is limited by the fact that we did not have a measure of surgical success or of reintegration experiences. Prior research has shown that depressive symptoms are more likely to persist among women who have incontinence post-surgery, and that severity of incontinence is associated with psychological distress [14, 35]. Additionally, women’s experiences of reintegration, including social support and stigma, greatly impact their psychological well-being [51, 52]. Finally, we acknowledge that we are limited by a relatively short 3-month follow up time, due to the program design. Future studies that are designed to understand the well-being of women following fistula repair should ideally include a longer follow-up, given evidence that physical and psychosocial health varies in the first 12 months following surgery [36]. Future studies would also benefit from a comprehensive measure of reintegration [51].

## Conclusion

In conclusion, this paper describes the mental health and well-being of Malian women presenting for obstetric fistula repair, and documents the impact of a routine mental health screening program on the practice of and policy surrounding fistula care in Mali. The findings from this study demonstrate the feasibility of integrating mental health screening into the fistula repair process. Moreover, the continued prevalence of moderate to severe depression in a subset of Malian fistula patients underscores a need for more robust mental health support for patients after surgery. Mental health interventions for fistula patients should be designed to address the psychosocial impacts and stigma associated with living with a fistula.
